# Diagnostic and Therapeutic Challenges of Oligosymptomatic Vesicovaginal Fistula in the Complex Case of Endometriosis

**DOI:** 10.3390/clinpract14020033

**Published:** 2024-03-12

**Authors:** Agnieszka A. Strojny, Arkadiusz Baran, Katarzyna Wiejak, Anna Scholz, Radosław B. Maksym

**Affiliations:** 1 1^st^ Department of Obstetrics and Gynecology, Centre of Postgraduate Medical Education, 02-004 Warsaw, Poland; a.strojny@szpitalzelazna.pl (A.A.S.); anna.scholz@cmkp.edu.pl (A.S.); 2 University Clinical Centre, Medical University of Warsaw, 02-097 Warsaw, Poland; katarzyna.wiejak@uckwum.pl

**Keywords:** endometriosis, adenomyosis, hysterectomy, partial cystectomy, vesicovaginal fistula, platelet-rich plasma

## Abstract

Endometriosis is a complex condition causing surgical challenges, sometimes leading to urogynecological complications, the diagnosis and treatment of which are not always obvious. We present a case of a 46-year-old woman with a history of severe endometriosis and adenomyosis who developed an oligosymptomatic vesicovaginal fistula (VVF) as a complication of surgery. The patient’s medical history included multiple surgeries for endometriosis, a cesarean section, and a laparoscopic hysterectomy. After the excision of the full-thickness infiltration of the urinary bladder, she experienced postoperative bowel obstruction treated by laparotomy. Subsequent urinary complications of bladder healing were eventually recognized as oligosymptomatic VVF. Symptoms of VVFs may vary, making a diagnosis challenging, especially when the lesion is narrow. Imaging techniques such as cystoscopy and cystography are helpful for diagnosis. The treatment options for VVFs range from surgical repair to conservative methods, like bladder catheterization, hormonal therapy, and platelet-rich plasma (PRP) injections, depending on the lesions’ size and location. In this case, the patient’s VVF was treated with PRP injections, a low-invasive method in urogynecology. PRP, known for its pleiotropic role, is increasingly used in medicine, including gynecology. The patient’s fistula closed after 6 weeks from the PRP session, highlighting the potential of this conservative treatment modality.

## 1. Introduction

Severe deep endometriosis remains a surgical challenge due to the high risk of complications, especially if numerous surgical procedures were previously performed on the patient. Although surgical operations should be performed completely and radically, in many situations, the lack of surgical skills in local centers and the reluctance to perform hysterectomies even on patients after childbearing is complete expose patients to further surgical treatment. An additional problem is the lack of therapeutic adherence to hormonal treatment after surgery. Each surgical intervention may cause peritoneal adhesions, enhanced by endometriosis infiltration in cases where deep nodules were not removed completely. Changes in anatomy can also occur as a result of improperly treated and progressive disease. Therefore, it is crucial to always consider the treatment strategy and timing of the surgical intervention carefully. Endometriosis may cause pelvic pain and infertility [1], but it may also have wider systemic implications [2]. Currently, there is no perfect treatment and, due to a high recurrence rate, the disease significantly reduces the quality of life of patients [1,2,3,4]. The diagnosis is made based on symptoms, physical examination, and imaging, but surgery can provide a definitive clinical and histopathological diagnosis. According to the latest recommendations, the treatment of endometriosis should be hormonal but surgical intervention is unavoidable in some cases [3]. Pharmacological therapy cannot be implemented, for example, in cases of severe adverse effects and patients wishing to conceive.

Adenomyosis remains a condition that falls within the spectrum of endometriosis. It is defined as an invasion of endometrial tissue into the myometrium and leads to an enlarged uterus, pain, and symptoms of abnormal uterine bleeding [5]. Often, patients with adenomyosis require a hysterectomy after completing childbearing, especially when symptoms do not respond to conservative treatment. A hysterectomy in the fifth decade of life, especially in patients burdened with a long history of untreated endometriosis and numerous non-radical surgeries, could be difficult and associated with possible long-term complications. Some consequences of surgery, however, are minimally symptomatic, making their management a diagnostic and therapeutic challenge. Further confounding the issue is that corrective measures should not bring additional hazards to these high-risk patients but should rather be well tailored to the problem.

## 2. Case Description

**Figure 1:**
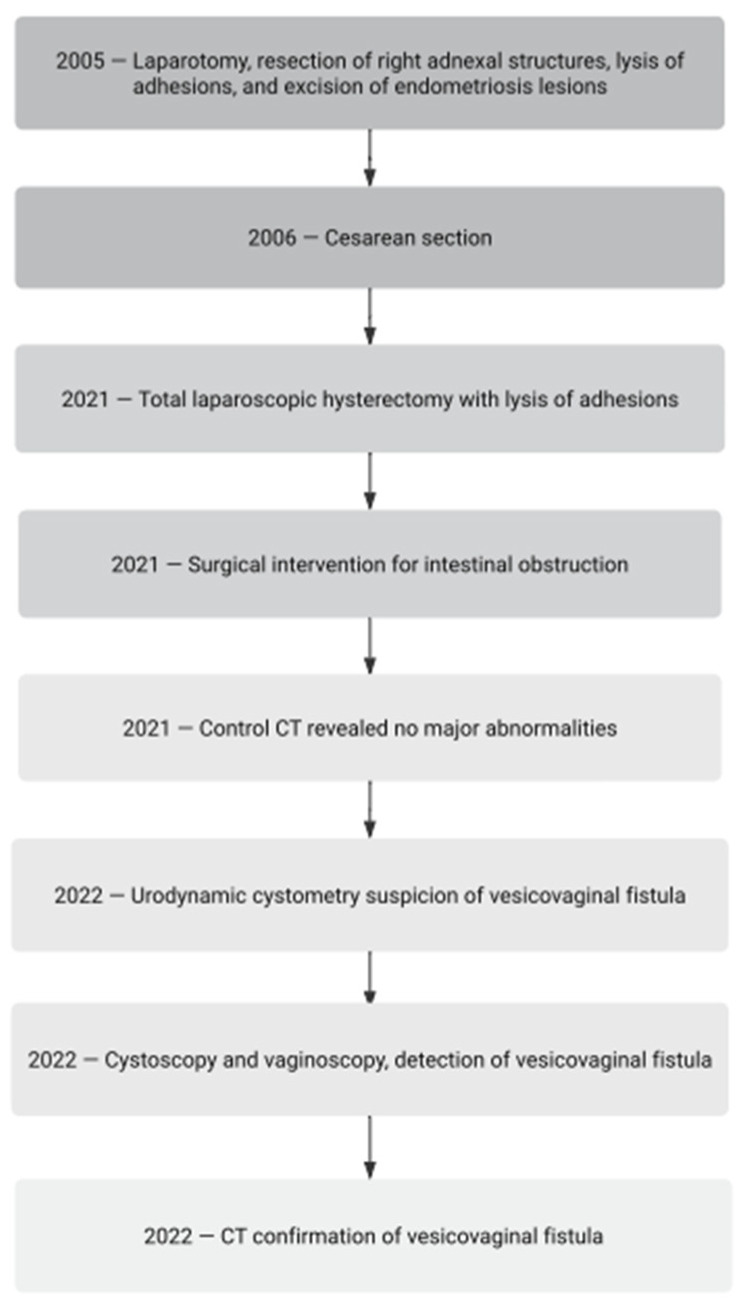
Chronological schematic representation of the main surgical procedures and diagnostic interventions performed.

A 46-year-old woman with endometriosis and adenomyosis and a history of numerous surgeries and medical interventions (Figure 1) was admitted to the gynecological department for the treatment of pain related to sexual intercourse, severe menstrual pain, and abnormal bleeding. The adenomyotic uterus and deep nodules were palpable. The symptoms were no longer responding to conservative treatment. The patient had been treated with 2 mg of dienogest monotherapy in the past after giving birth. She discontinued the drug due to poor tolerability associated with a depressed mood and chronic genital spotting. Continuous hormone therapy was then attempted with a formulation containing 0.03 mg of ethinylestradiol and 2 mg of dienogest (combined oral contraceptive). She tolerated the therapy well but did not achieve full improvement in her complaints. She opted not to be treated with a levonorgestrel-releasing therapeutic system (52 mg LNG-IUD).

In the past (2005), the patient underwent a laparotomy because of a right ovarian endometrioma, including the removal of a right adnexa, the lysis of adhesions, and the excision of deep endometriosis lesions. Following these procedures and subsequent management, she experienced a successful pregnancy. The course of the pregnancy was normal. The birth of a 4150 g male newborn was realized via cesarean section in 2006. An urgent cesarean section was performed at the 16th hour of natural childbirth, after 2 h of the second period of labor due to threatening fetal asphyxia diagnosed on the basis of late decelerations in the cardiotocographic recording. The course of the cesarean section and puerperium was not complicated. This was the only pregnancy and birth in her life.

In August 2021, the patient qualified for a total laparoscopic hysterectomy with the excision of the left oviduct. During the operation, massive adhesions of the bowel to the right side of the uterine body and the full-thickness infiltration of the urinary bladder were revealed. Adhesions of the large intestine were released, and the uterus and left oviduct were excised. In order to excise the bladder tumor, a partial cystectomy was performed. Briefly, during resection, a full-thickness 4 cm opening of the bladder wall was created, and then the bladder was sutured in two layers during laparoscopy. After surgery, a double J (DJ) catheter was inserted into the left ureter, and the bladder was catheterized with a Foley catheter. A histopathological examination confirmed the diagnosis of endometriosis and adenomyosis. 

One week following the prior surgical procedure, the patient arrived at the surgical ward with heightened abdominal discomfort, accompanied by symptoms indicative of gastrointestinal obstruction such as nausea, vomiting, severe bloating, peritoneal signs, and constipation. Upon the diagnosis of gastrointestinal obstruction, surgical intervention was deemed necessary. Postoperative monitoring revealed an absence of surgical complications. Subsequently, as there was no longer a requirement for catheterization, both catheters were removed. The surgical operation consisted of a thorough revision of the abdominal cavity and the finding of a volvulus of the small intestine. The twisted hypermobile loop of the small intestine was then translocated to its normal position, without the need for resection. A subsequent computed tomography (CT) scan exhibited no significant abnormalities. With the patient exhibiting a favorable general condition, the implementation of an easily digestible diet during hospitalization ensued, culminating in discharge from the ward. In February 2022, the patient began to report abundant vaginal discharge at night, which disappeared during daytime activities. Initially, a general urine test was performed, which showed no abnormalities. Subsequently, an abdominal ultrasound was performed that revealed normal voiding capacity and a normal pelvicalyceal system. Due to the above symptoms, the patient was initially taking mirabegron, which she discontinued after 3 months due to constipation. She was then treated with solifenacin, with a good tolerance and satisfactory effect for some time.

**Figure 2:**
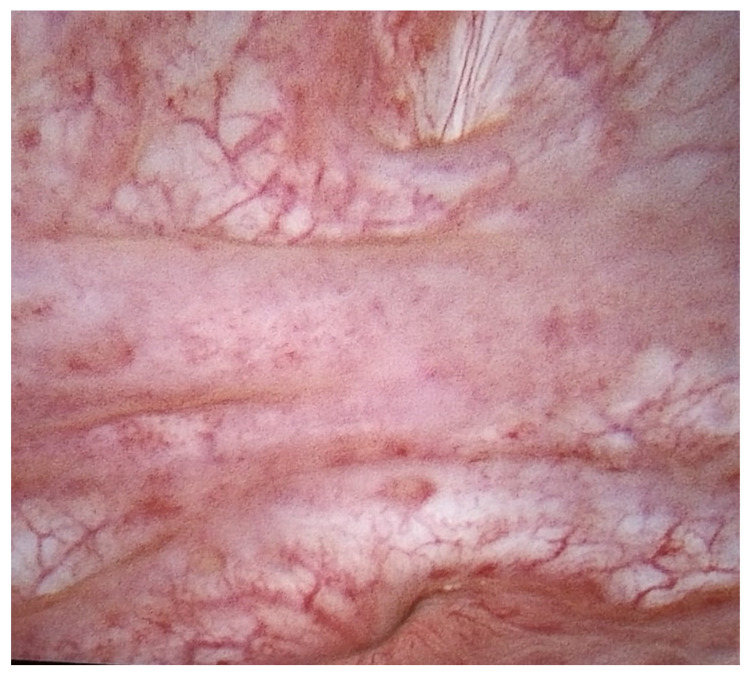
Cystoscopic view of oligosymptomatic vesicovaginal fistula.

In August 2022, the issue of urinary incontinence was raised again by the patient. Urodynamic cystometry was conducted, revealing low-amplitude detrusor overactivity. During the performance of the Valsalva and coughing maneuvers at bladder volumes of 250 mL and 350 mL, no urinary leakage was observed. However, at volumes of 350 mL and 450 mL, a slight leakage was noted. The suspicion of a vesicovaginal fistula was identified. After two weeks, due to emerging dysuric symptoms and developing urinary incontinence, a cystoscopy was performed during which a urinary bladder with an increased vascular pattern was visualized, with the area above the urinary bladder triangle distorted and an opening of the fistula approximately 2 cm from the left ureter outlet (Figure 2). A narrow catheter was inserted into the opening, and communication with the vagina was confirmed. Then, a vaginoscopy was performed, revealing an area of the fistula opening with a diameter of approximately 2 mm at the apex of the vagina.

In September 2022, a CT scan with contrast was performed, which revealed a space within the vaginal apex with contrast accumulation in the empty phase, which was located posterior to the urinary bladder, as well as single gas bubbles in the urinary bladder. A urodynamic examination was also performed, which revealed low-amplitude detrusor overactivity and vaginal urine leakage during attempts to fill the bladder.

The diagnosis of a vesicovaginal fistula was made. Inflammation associated with postoperative gastrointestinal obstruction and the early removal of catheters prevented the bladder opening from healing properly. The resulting fistula was small enough to produce discrete symptoms. Initially, muscle tension during daily activities closed the fistula gates and made diagnosis difficult. Only a decrease in muscle tension while at rest resulted in urine leakage, which was initially interpreted as vaginal discharge. Symptoms began to worsen because of increasingly frequent bladder infections due to bacteria infiltrating the vagina. The fistula was treated with a single injection of platelet-rich plasma (PRP), and a urinary bladder catheter was inserted again. We used autologous PRP obtained in a closed system RegenKit^®^ BCT-T (REF# RK-BCT-T, Regen Lab SA, Le Mont-sur-Lausanne, Switzerland). After collecting 10 mL of venous blood from the elbow flexure in a test tube containing separation gel, the test tube was centrifuged at an overload of 1500 *g* for 10 min. The plasma was then collected along with the platelet pellet and mixed. Subsequently, 4 × 1 mL of PRP was injected once into the fistular area on the four sides of the fistula (around the fistula hole). The injection was performed during a cystoscopy performed under brief general intravenous anesthesia. A Foley catheter was left in the bladder. After 6 weeks of treatment, the vesicovaginal fistula closed, and the catheter was removed from the urinary bladder. We confirmed the healing of the fistula during a subsequent cystoscopy. During further observation, no symptoms of a fistula were noticed for 12 months of follow-up, and the treatment was considered completed.

## 3. Discussion

A vesicovaginal fistula (VVF) represents an abnormal connection between the urinary bladder and the vagina. In developed countries, the majority of VVF cases occur as a complication of cesarean sections or other gynecological surgeries. Less frequently, a VVF follows obstetric complications, diseases of the lesser pelvis, radiation therapy, or prolonged indwelling urinary catheterization [6,7]. A VVF is a relatively common urogynecological complication, yet it sometimes remains challenging to diagnose, often presenting diagnostic and therapeutic dilemmas. Clinical manifestations of VVFs can vary, complicating prompt detection. The symptoms of complex fistulas may include cyclical hematuria (menstruation), amenorrhea, urinary leakage from the vagina with or without urinary incontinence, recurrent urinary tract infections accompanied by low-grade fever, secondary infertility, first-trimester miscarriages, cystitis, hematuria, and pruritus vulvae [3,4,8,9,10]. Various imaging procedures have proven useful in diagnosis. Cystoscopy, cystography, and hysterosalpingography (HSG) play a pivotal role in diagnosing patients with a VVF. Additional methods include contrast-enhanced computed tomography (CT), magnetic resonance imaging (MRI), and transvaginal ultrasound [7,10,11].

Increasingly, the literature describes various cases of this complication and considers different therapeutic options. For large fistulas, the primary treatment method is surgical repair through the transvaginal Latzko procedure or surgical closure using the Martius flap [11,12]. For small fistulas, various therapeutic possibilities have been indicated in literature. In many described cases, surgical treatment remains key, with various approaches recommended, including transperitoneal, transvesical, and transvaginal, along with different surgical repair techniques [10,13].

However, the conservative treatment of small fistulas is progressively proposed, using bladder catheterization, hormonal therapy, platelet-rich plasma (PRP) injections, or the cystoscopic fulguration of VVFs [10,12,14]. Conservative methods are particularly desirable in cases where patients have undergone multiple surgeries, and another intervention is associated with an extremely high risk of complications. These methods have shown good treatment outcomes without a need for further surgical intervention. In our case, the vesicovaginal fistula occurred as a complication of a partial cystectomy for endometriosis and subsequent surgery due to bowel obstruction. Gastrointestinal surgery along with inflammation and the non-planned removal of a Foley catheter resulted in bladder healing impairment. The developing fistula was small enough to produce minor and nonspecific symptoms. In the first phase, muscle tension during daily activities compressed the fistula gates and made diagnosis awkward. At night, resting muscles relaxed and urine leakage occurred. Initially, the volume was so small that it was interpreted as vaginal discharge. Symptoms began to worsen because of increasingly frequent bladder infections by bacteria infiltrating the vagina. The VVF was diagnosed during cystoscopy and vaginoscopy; in this case, the fistula had a diameter of approximately 2 mm. Due to the small size of the fistula, treatment with PRP injections was employed with success.

Platelet-rich plasma (PRP) is the liquid fraction of peripheral blood containing a high concentration of platelets, growth factors, and bioactive components [15]. PRP plays a positive role in tissue healing by stimulating angiogenesis, cell migration and proliferation, and extracellular matrix production [15]. PRP has various applications in medicine, particularly in accelerating the healing process of musculoskeletal injuries, such as with cartilage or ligaments [15]. In gynecology, PRP therapy is a promising method for treating various conditions, including urogenital disorders, wound healing, and vulvar atrophy [16]. PRP injections are a novel therapeutic method for various conditions that are also applied in gynecology, including the treatment of vesicovaginal fistulas. PRP is administered vaginally. Recently, 15 cases of VVFs treated with PRP injections were described. PRP injections were usually followed by the Latzko method. In one of the reported cases, the fistula closed spontaneously after PRP application only [17]. A similar effect was achieved in our patient, whose fistula closed 6 weeks after the PRP injection and bladder catheterization.

## 4. Conclusions

Vesicovaginal fistulas cause symptoms that significantly reduce the quality of life of patients and still constitute a major therapeutic problem. So far, the main method of treatment has been surgery, but more conservative methods of treatment are being sought, especially in cases where numerous surgeries were carried out before. Some VVFs, however, are minimally symptomatic, making their management a diagnostic and therapeutic challenge. A further confusing issue is that corrective measures should not bring additional risks to these demanding patients but should rather be well personalized to the case. One of the promising methods is the injection of platelet-rich plasma, which enables the healing of small fistulas. Nonetheless, further research is necessary to precisely determine the indications, conditions, and full applicability of the method.

## Data Availability

Data confirming the results presented can be obtained from the corresponding author upon any reasonable request, considering the problems associated with the protection of patients’ personal data.
